# Eosinophilic Duodenitis and Jejunitis with Ascites and Peripheral Eosinophilia: A Diagnostic Challenge—Case Report

**DOI:** 10.3390/life16081262

**Published:** 2026-07-30

**Authors:** Oana-Bogdana Barboi, Constantin Simiras, Radu-Alexandru Vulpoi, Diana-Elena Floria, Vadim Rosca, Delia Gabriela Ciobanu-Apostol, Corina Hincu, Vasile-Liviu Drug

**Affiliations:** 1 Faculty of Medicine, “Grigore T. Popa” University of Medicine and Pharmacy, 700115 Iasi, Romania; oany_leo@yahoo.com (O.-B.B.); vulpoi.radu@yahoo.ro (R.-A.V.); iovdiana95@gmail.com (D.-E.F.); vroshca94@gmail.com (V.R.); delia.ciobanu@umfiasi.ro (D.G.C.-A.); vasidrug@email.com (V.-L.D.); 2 Institute of Gastroenterology and Hepatology, Saint Spiridon Emergency County Hospital, 700111 Iasi, Romania; 3 Department of Morpho-Functional Sciences–Pathology, Saint Spiridon Emergency County Hospital, 700111 Iasi, Romania; 4 Department of Radiology, Saint Spiridon Emergency County Hospital, 700111 Iasi, Romania; corinaelenahincu@gmail.com

**Keywords:** eosinophilic jejuno-duodenitis, eosinophilia, eosinophilic infiltration

## Abstract

**Background:** Eosinophilic jejuno-duodenitis is a rare inflammatory disorder characterized by eosinophilic infiltration of the gastrointestinal tract, with highly variable clinical presentations. Mucosal eosinophilic duodenitis and jejunitis associated with ascites is uncommon and may mimic inflammatory or neoplastic conditions. **Case Presentation:** A 26-year-old male presented with persistent left-sided abdominal pain without bowel habit changes. Laboratory investigations revealed only slight inflammatory syndrome and peripheral eosinophilia. Contrast-enhanced abdominopelvic computed tomography demonstrated minimal ascites and circumferential wall thickening with contrast enhancement of duodenal and jejunal loops in the left flank. Given the nonspecific imaging findings, differential diagnoses included Crohn’s disease, intestinal lymphoma, and infectious enteritis. Endoscopic evaluation with histopathological analysis confirmed dense eosinophilic infiltration of the duodenal and jejunal mucosa, establishing the diagnosis of eosinophilic jejuno-duodenitis after exclusion of secondary causes. **Management and Outcome:** The patient was treated with budesonide, with rapid clinical improvement and resolution of inflammatory markers. Follow-up magnetic resonance enterography performed three months after initiation of treatment demonstrated complete resolution of bowel wall thickening and disappearance of the minimal ascites. **Conclusions:** Eosinophilic jejuno-duodenitis should be considered in young patients presenting with small bowel thickening, ascites, and peripheral eosinophilia. Awareness of this entity is essential to avoid misdiagnosis and unnecessary invasive procedures.

## 1. Introduction

Eosinophilic gastroenteritis (EGE) is a rare and heterogeneous inflammatory disorder [1,2,3] characterized by eosinophilic infiltration of the gastrointestinal tract in the absence of identifiable secondary causes, such as parasitic infections, drug reactions, systemic diseases, or malignancy [1]. The condition may involve any segment of the digestive tract, most commonly the stomach (40–70%) and duodenum or ileum (10–25%) [4], rarely the colon or jejunum [5]. It is traditionally classified according to the depth of wall involvement into mucosal, muscular, and serous forms, each associated with distinct clinical manifestations.

According to current consensus recommendations, eosinophilic gastrointestinal disease is classified according to the anatomical segment involved. In the present case, the diagnosis was consistent with eosinophilic duodenitis with eosinophilic jejunitis, based on compatible clinical manifestations, histopathological evidence of marked eosinophilic infiltration in duodenal and jejunal biopsies, exclusion of secondary causes of eosinophilia, and the subsequent favorable response to corticosteroid therapy.

The clinical presentation of EGE is highly variable and often nonspecific, ranging from abdominal pain, diarrhea, and malabsorption (in mucosal disease) to obstructive symptoms (in muscular involvement) and ascites (in the serous form). Peripheral eosinophilia is found in half of the cases, further complicating the diagnostic process [6,7]. Due to the lack of pathognomonic features, EGE remains a diagnostic challenge and is frequently misdiagnosed as more common entities, particularly Crohn’s disease, infectious enteritis, or gastrointestinal malignancies.

Involvement of the jejunum, especially in combination with duodenal disease, is uncommon and poorly represented in the literature, with most available data derived from isolated case reports or small case series. Moreover, the association of small bowel involvement with ascites suggests possible serous disease, a less frequent but clinically significant presentation that may further obscure the diagnosis by mimicking peritoneal carcinomatosis or tuberculous peritonitis [8].

Herein, we report a case of eosinophilic jejuno-duodenitis in a young adult presenting with left-sided abdominal pain, peripheral eosinophilia, and ascites, highlighting the diagnostic challenges and the importance of considering this rare entity in the differential diagnosis of small bowel thickening.

## 2. Case Presentation

We report the case of a 26-year-old male who presented to our Gastroenterology department with persistent left-sided abdominal pain for three months, but with no history of bowel habit changes or other symptoms. At presentation, the patient was in good overall condition. Physical examination revealed mild abdominal tenderness without signs of peritonitis. Laboratory investigations showed only slightly elevated inflammatory syndrome (C-reactive protein level 1.2 mg/dL, with normal range 0–0.5 mg/dL). The total leukocyte count was 10.14 × 10^3^/μL, with an absolute eosinophil count of 2.05 × 10^3^/μL (20.2%). Hemoglobin levels and fecal calprotectin were within the normal reference range, making active inflammatory bowel disease less likely.

A detailed medical history revealed no regular medication, dietary supplements, recent travel, consumption of raw or undercooked food, or occupational exposures associated with eosinophilia. Stool cultures and repeated parasitological examinations were negative. Clinical evaluation and laboratory investigations identified no evidence of celiac disease, inflammatory bowel disease, autoimmune or vasculitic disorders, hematologic malignancy, solid-organ malignancy, drug-induced eosinophilia, or hypereosinophilic syndrome. Therefore, secondary causes of gastrointestinal eosinophilia were considered unlikely.

Serum total protein was 6.20 g/dL and serum albumin was 4.38 g/dL, both within the normal reference range, making hypoproteinemia an unlikely explanation for the ascites.

**Figure 1:**
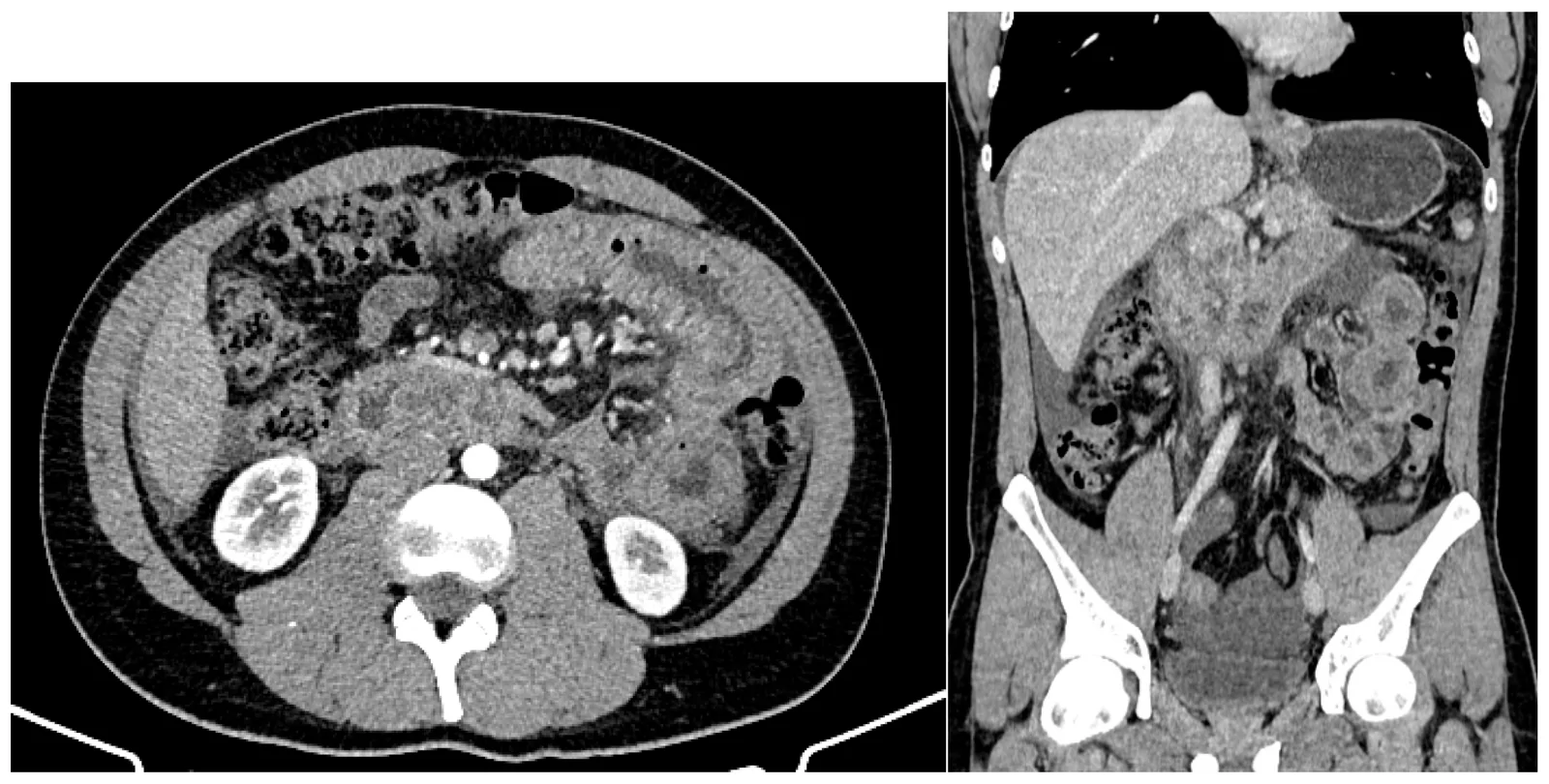
Contrast-enhanced abdomino-pelvic CT scan (axial and coronal reconstructions). Thickening of the duodenal and jejunal walls and intraperitoneal fluid.

Abdominal ultrasound demonstrated ascites without any additional pathological findings. Further, contrast-enhanced abdominal–pelvic computed tomography was performed. Circumferential wall thickening of duodenal and jejunal loops located in the left flank, with moderate contrast enhancement, was identified. No signs of bowel obstruction or perforation were observed. CT also demonstrated a small volume of free intraperitoneal fluid distributed within the dependent peritoneal recesses, consistent with minimal ascites, without evidence of a loculated fluid collection or abscess (Figure 1).

**Figure 2:**
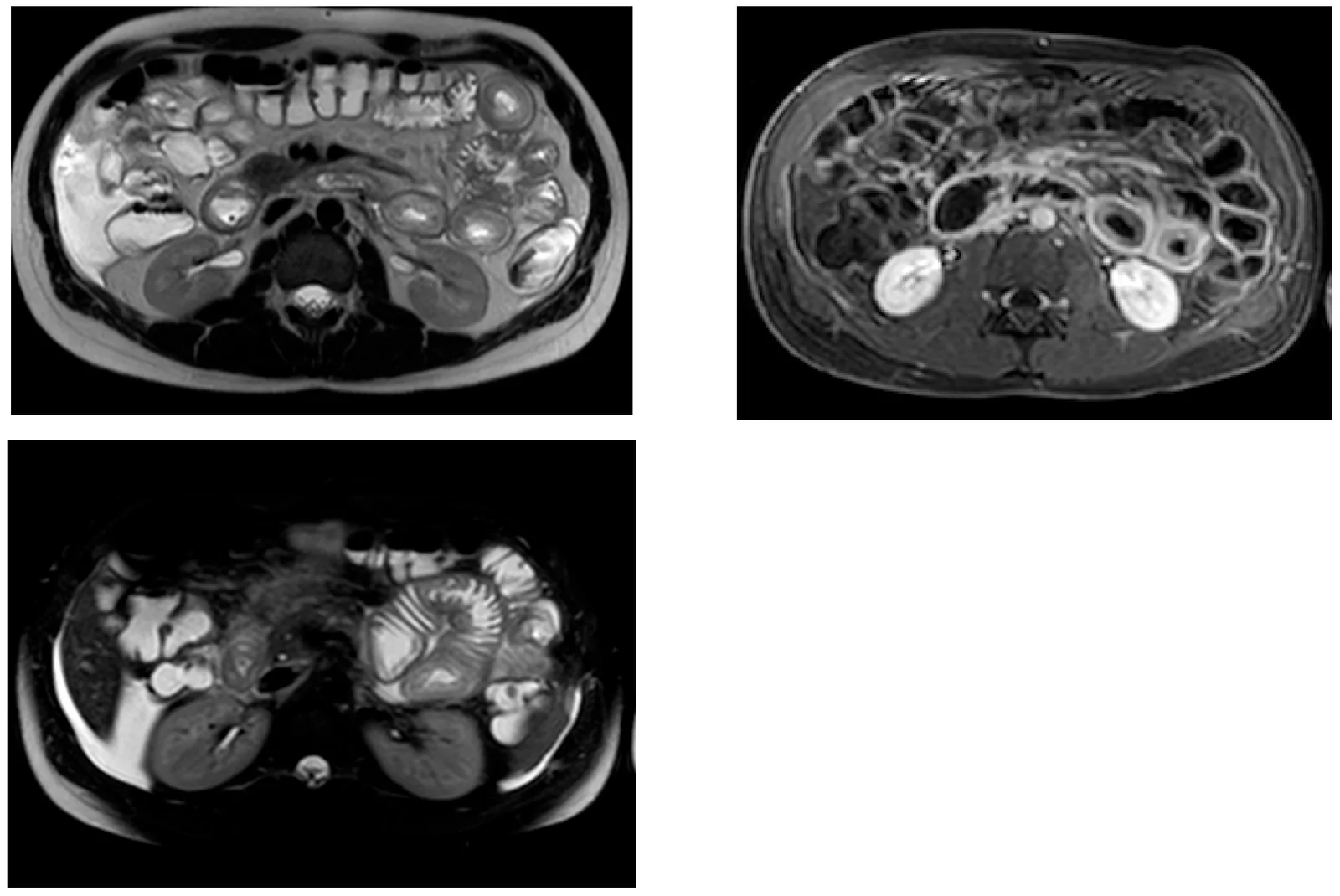
Contrast-enhanced abdominal–pelvic MRI. T2 and post-contrast T1-weighted sequence in the axial plane: thickened jejunal loops and intraperitoneal fluid.

Additional investigations were performed to exclude infectious and structural lesions. Magnetic resonance enterography revealed, in addition to slight ascites, inflammatory changes in the duodenum and jejunum mucosa, with preserved wall stratification and multiple homogeneous solid lymph nodes (maximum short-axis diameter, 8 mm) at the root of the adjacent mesentery (without vascular hyperemia suggestive of a “comb sign”) (Figure 2). The lymph nodes were interpreted as reactive, without radiological features suggestive of malignancy. The imaging findings were not suggestive of active inflammatory bowel disease. Together with the normal fecal calprotectin level, Crohn’s disease was unlikely; however, they were not sufficient to definitively exclude inflammatory bowel disease.

**Figure 3:**
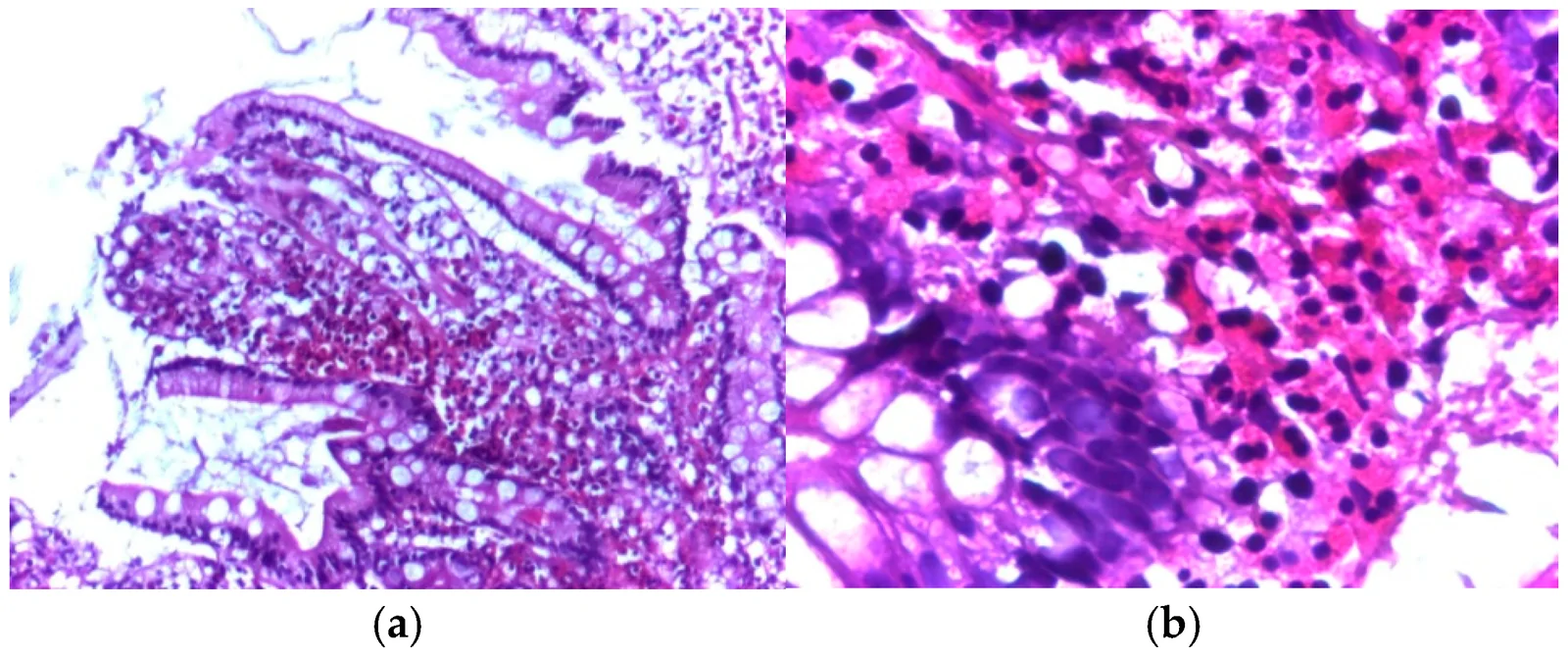
Findings on histopathological examination: (**a**) Eosinophil infiltration in the lamina propria of the duodenum (hematoxylin–eosin staining, magnification × 200). (**b**) Eosinophil infiltration in the lamina propria of the jejunum (hematoxylin–eosin staining, magnification × 400).

Considering these findings, push enteroscopy with multiple biopsies and histopathological analysis of jejunal biopsies were needed. Duodenal and jejunum erythematous mucosa was found, with the esophagus and stomach being unremarkable. Histopathological examination of the duodenal and jejunal biopsy specimens demonstrated dense mucosal infiltration by eosinophils, with peak counts exceeding 200 eosinophils per high-power field (HPF) in both locations. The eosinophilic infiltrate predominantly involved the lamina propria, without histopathological features suggestive of Crohn’s disease, celiac disease, infectious enteritis, or malignancy (Figure 3). No eosinophilic infiltration or related abnormalities were identified within the esophageal or gastric mucosa.

In the present case, serum total protein and albumin concentrations were within the normal reference range, making hypoproteinemia and protein-losing enteropathy less likely explanations for the ascites. Although the presence of free ascites may suggest inflammatory serosal involvement, this could not be confirmed because neither ascitic fluid analysis nor full-thickness biopsy was performed. Therefore, the exact mechanism underlying ascites formation remains uncertain and should be interpreted with caution.

**Figure 4:**
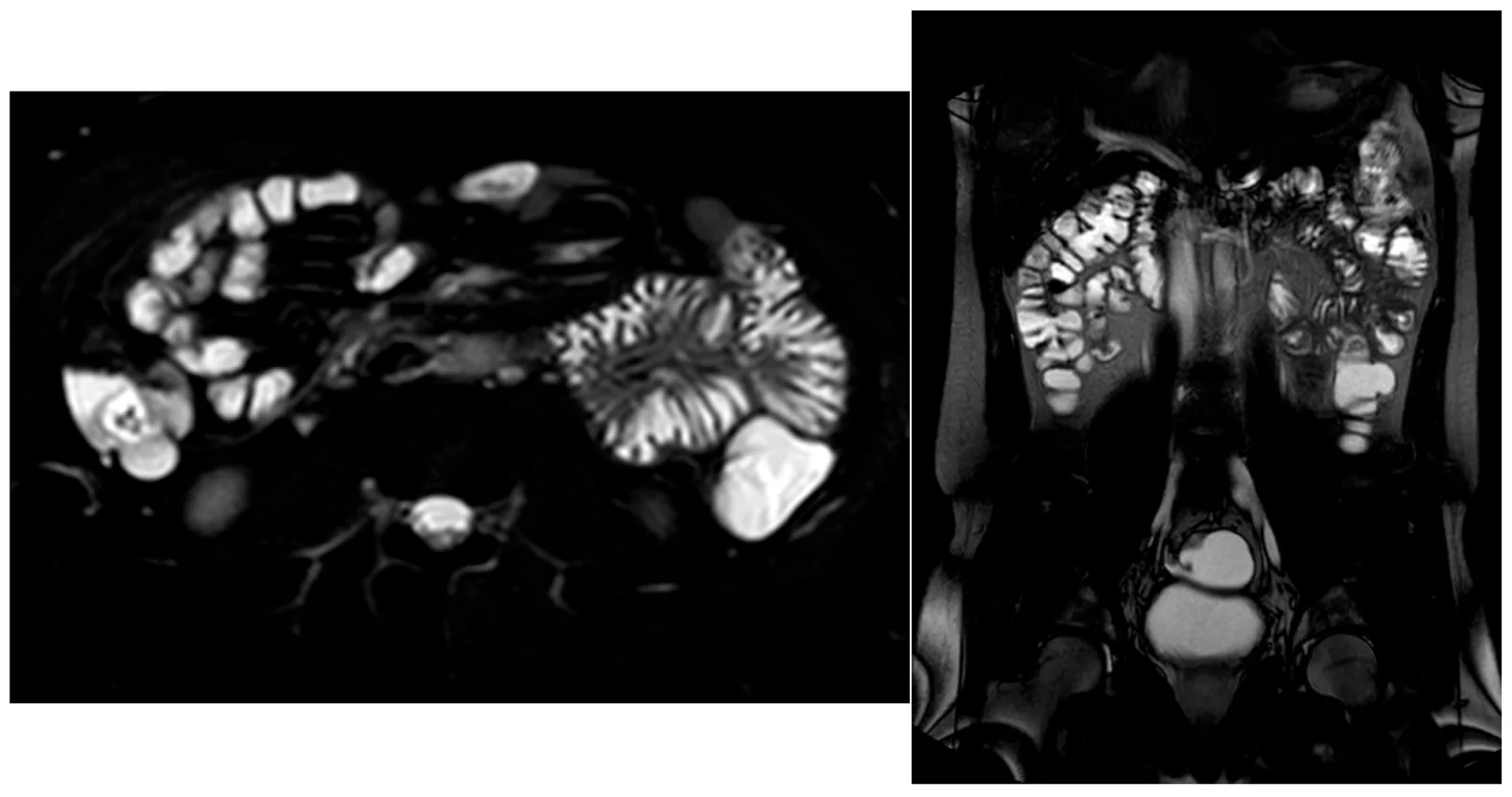
Follow-up magnetic resonance enterography showing complete resolution of the jejunal wall thickening and disappearance of ascites. Images include coronal and axial T2-weighted sequences.

The patient was treated with oral budesonide (Budenofalk^®^) using a tapering regimen consisting of 9 mg/day for the first month, followed by 6 mg/day for one month and 3 mg/day for a further month. Budesonide was selected because of its favorable safety profile and its established efficacy in eosinophilic gastrointestinal disorders with predominant mucosal involvement. The patient demonstrated excellent adherence to treatment and reported no adverse effects. At the 1-month follow-up visit, abdominal pain had completely resolved, while peripheral eosinophilia and inflammatory markers had normalized (eosinophil count of 0.25 × 10^3^/μL (3.5%) and C-reactive protein level of 0.18 mg/dL). Follow-up magnetic resonance enterography performed after 3 months demonstrated complete resolution of the duodenal and jejunal wall thickening and disappearance of ascites (Figure 4). Because the patient achieved complete clinical, laboratory, and radiological remission, repeat enteroscopy with biopsy was not performed; therefore, histological remission could not be confirmed.

## 3. Discussion

Eosinophilic gastrointestinal disorders represent a heterogeneous group of conditions characterized by eosinophilic infiltration in the absence of secondary causes. Small bowel involvement is relatively uncommon, and isolated or predominant jejunal disease remains rarely reported, in general, complicated by an acute surgical abdomen [9]. Colonic locations of EGE are also rare, with most cases being ileocolic forms that can pose diagnostic challenges with Crohn’s disease [5]. Peritoneal involvement, which is uncommon (occurring in less than 10% of cases), leads to ascites that is often transient in nature. Preceding abdominal bloating is a nonspecific symptom. This subtype is typically associated with marked peripheral eosinophilia [7].

Regarding disease distribution, our case is particularly notable for isolated involvement of the proximal small intestine, namely the jejuno-duodenal region, while the remaining segments of the gastrointestinal tract were unaffected.

Although eosinophilic gastrointestinal diseases have been increasingly recognized in recent years, isolated eosinophilic involvement of both the duodenum and jejunum associated with ascites remains exceptionally uncommon in adults [4]. Most published reports describe eosinophilic gastroenteritis with predominant gastric or diffuse small bowel involvement, while concomitant duodenal and jejunal disease presenting with peripheral eosinophilia and ascites has only been sporadically documented. Furthermore, ascites is more frequently associated with serosal disease, whereas our patient presented with histologically confirmed mucosal eosinophilic inflammation associated with ascites in the absence of histological confirmation of serosal involvement. This case therefore highlights the diagnostic complexity of eosinophilic gastrointestinal disease, particularly when clinical and radiological findings overlap with other inflammatory disorders, such as Crohn’s disease. In addition, the favorable clinical, laboratory, and radiological response to oral budesonide further supports its role as an effective therapeutic option for selected patients with mucosal eosinophilic gastrointestinal disease.

Eosinophilic infiltration of the gastrointestinal wall may present either as isolated gastroenteritis or as a manifestation of essential hypereosinophilic syndrome, involving cardiac, pulmonary, and neurological systems [10]. The clinical presentation of EGE disorders is variable, with symptom patterns depending on the affected layer of the digestive wall. When the inflammatory infiltrate is predominantly confined to the mucosal layer, patients typically present with nausea, vomiting, abdominal pain, diarrhea, malabsorption, and protein-losing (exudative) enteropathy. When the muscular layer is involved, obstructive symptoms are typically predominant. In contrast, isolated or predominant serous involvement is less common and is characterized by eosinophil-rich ascites and, in some cases, associated pleural effusion [11].

Our case is notable for several atypical features. First, the duodenal and jejunal localization of the disease, in the absence of colonic involvement, is unusual. Second, the patient presented with left-sided abdominal pain without diarrhea or malabsorption, which are commonly described in mucosal forms. Third, the presence of ascites raised the possibility of serosal involvement. However, serosal eosinophilic disease could not be confirmed because diagnostic paracentesis was not feasible due to the small volume of ascitic fluid, and no full-thickness biopsy was obtained. Therefore, the association between ascites and eosinophilic inflammation should be interpreted cautiously, and possible serosal involvement remains hypothetical. Unfortunately, the amount of ascites did not permit paracentesis, which would have been of great diagnostic value. Fourth, peripheral eosinophilia supported the diagnosis (but is not universally present), along with the lack of patient atopic background, further contributing to diagnostic variability.

In general, computed tomography provides limited additional diagnostic value, often revealing only nonspecific fold thickening [1]. In our case, the radiological findings were also nonspecific and included Crohn’s disease among the differential diagnostic considerations together with intestinal lymphoma and infectious enteritis. Although fecal calprotectin was within the normal range and magnetic resonance enterography did not demonstrate imaging features suggestive of active Crohn’s disease, a complete ileocolonoscopic assessment with terminal ileal biopsies was not performed. Therefore, Crohn’s disease remained a differential diagnostic consideration rather than being formally excluded.

The coexistence of ascites could have further biased the diagnostic approach toward malignancy or tuberculosis. Therefore, histopathological confirmation of the jejunal specimen obtained by endoscopy remained essential. Histopathological examination confirmed dense mucosal eosinophilic infiltration, with peak counts exceeding 200 eosinophils per high-power field in both the duodenal and jejunal biopsy specimens, establishing the diagnosis after exclusion of secondary causes. In the majority of cases, endoscopic examination shows nonspecific findings, like erythematous, ulcerative or nodular mucosa, and sometimes peptic ulcers [1]. This is also the case of our patient, in whom endoscopy and enteroscopy revealed erythematous jejunal and duodenum mucosa.

Budesonide was preferred over systemic corticosteroids because of its high topical anti-inflammatory activity, extensive first-pass hepatic metabolism, and favorable safety profile, making it an appropriate first-line treatment for mucosal eosinophilic gastrointestinal disease. The rapid response to oral budesonide observed in our patient is consistent with previously reported data [9,12], supporting the role of corticosteroids as first-line therapy, especially for the forms of the disease with predominant mucosal involvement. Corticosteroid treatment led in our patient to a striking improvement across clinical symptoms, laboratory parameters, and imaging findings. However, relapse rates remain significant, and long-term follow-up is warranted [9].

Recent advances in the understanding of eosinophilic gastrointestinal diseases have highlighted the central role of type-2 immune responses in disease pathogenesis. Epithelial-derived alarmins, including thymic stromal lymphopoietin (TSLP), IL-25, and IL-33, promote activation of type 2 innate lymphoid cells (ILC2s) and Th2 lymphocytes, leading to increased production of IL-4, IL-5, and IL-13 [13,14]. These cytokines drive eosinophil recruitment, activation, and survival within the gastrointestinal mucosa, contributing to tissue inflammation and remodeling. This evolving understanding of the underlying immunological mechanisms has also provided the rationale for the development of targeted biological therapies in eosinophilic gastrointestinal diseases, although corticosteroids remain the standard first-line treatment for most patients.

## 4. Strengths and Limitations

The main strength of this case report lies in the comprehensive multidisciplinary diagnostic approach, including cross-sectional imaging, push enteroscopy with histopathological confirmation, exclusion of secondary causes of eosinophilia, and the favorable clinical, laboratory, and radiological response to budesonide therapy. In addition, this report highlights an uncommon presentation of eosinophilic duodenitis and jejunitis associated with ascites, emphasizing the importance of considering eosinophilic gastrointestinal disease in the differential diagnosis of inflammatory small bowel disorders.

The present report also has several limitations. As a single-case observation, the findings cannot be generalized. Ascitic fluid analysis and full-thickness intestinal biopsy were not performed; therefore, serosal involvement could not be confirmed, and the precise mechanism underlying ascites remains uncertain. Furthermore, histological remission was not confirmed by repeat endoscopy, and long-term follow-up after treatment completion was limited.

## 5. Conclusions

Eosinophilic jejunal–duodenal involvement should be considered in the differential diagnosis of small bowel wall thickening associated with ascites, particularly in young patients without typical features of inflammatory bowel disease or infection. Early recognition of eosinophilic duodenitis and jejunitis may help prevent unnecessary surgery, delayed diagnosis, or inappropriate treatment for presumed inflammatory bowel disease. Nevertheless, endoscopic evaluation with histopathological examination remains essential for establishing the diagnosis.

## Data Availability

All data supporting the findings of this study are available from the corresponding author upon reasonable request.
